# New insights into the potential introduction pathways of *Callinectes sapidus* Rathbun, 1896 (Decapoda, Portunidae) in European waters

**DOI:** 10.3897/BDJ.14.e215033

**Published:** 2026-10-23

**Authors:** Elena Ortega Jimenez, Enrique González Ortegón, Jose A Cuesta

**Affiliations:** 1 ICMAN-CSIC, Puerto Real, Spain ICMAN-CSIC Puerto Real Spain

**Keywords:** COI mitochondrial DNA, Guadalquivir Estuary, haplotype diversity, invasive species, invasion history, population genetics, sampling effort

## Abstract

Two new COI haplotypes of the invasive *Callinectes sapidus* were reported for the first time in the western Iberian Peninsula and one of them in Europe, providing new insights into the origin of this species' invasion in European waters. The new haplotypes were recorded at very low frequencies (< 1%) in the Guadalquivir Estuary during 2021 and 2022 and correspond to those reported in the Gulf of Mexico and Brazil, respectively. These findings indicate new possible introduction pathways from these regions. In addition, the Gulf of Mexico haplotype found in the Guadalquivir Estuary differs by only five mutations from haplotypes recently observed in Tunisia, suggesting a potential genetic link between these populations. The population of the Guadalquivir Estuary was mainly represented by the CSWM1 haplotype (66%), followed by the CSWM2 (32%), aligning with the westward expansion pattern observed in other west Mediterranean and east Atlantic populations.

## Introduction

Identifying the source of introduced populations helps predict dispersal routes, modes of introduction and improves the management and control of invasive species (Muirhead et al. 2008). Mitochondrial molecular biomarkers, such as COI, have been used to identify the potential genetic links between introduced and source populations in order to reconstruct the origin and invasion routes of non-native species (Geller et al. 2010, Rodrigues et al. 2017, Lamkhalkhal et al. 2026) The invasive blue crab *Callinectes sapidus* (Rathbun 1896) is native to the east coast of the Americas and it was first introduced to Europe in the 1900s (Holthuis 1961, Mancinelli et al. 2013) becoming established across eastern Mediterranean populations during the 1950s (Enzenroß et al. 1997).

The initial appearance of this species close to ports, together with its extended larval duration, suggests that the species was principally introduced by ballast water in Europe (Nehring 2011). However, there is additional evidence of intentional introductions for aquaculture purposes along the coasts of Turkey (Nehring 2011, Schubart et al. 2023).

In the native area, the species exhibits a high genetic structure along the Atlantic coast, with a total of 174 haplotypes grouped in three lineages associated with geographical latitude (1: North America and Gulf of Mexico, 2: Caribbean and 3: South America) (Windsor et al. 2019). Initial studies across Mediterranean and Atlantic populations revealed a strong founder effect within lineage 1, detecting only 12 haplotypes, with two dominant haplotypes, CSWM1 and CSWM2, being the most prevalent (Windsor et al. 2019, González-Ortegón et al. 2022, Schubart et al. 2023).

The other 10 haplotypes were specific to Turkey (Aegean and Black Seas) and differ by two to ten mutations from the North American dominant haplotype (H34) and CSWM1 and CSWM2 (Schubart et al. 2023). The higher haplotype richness and the genetic divergence observed in Turkey compared to other blue crab Mediterranean populations have been related to the longer invasion history in the region, as well as to intentional founding events for aquaculture stocks (Schubart et al. 2023, Locci et al. 2024).

The dominance of the two widespread haplotypes CSWM1 and CSWM2 in most Atlantic and western Mediterranean populations (Besbes et al. 2024) likely reflects a genetic founder effect associated with a westward expansion from eastern Mediterranean populations (González-Ortegón et al. 2022, Schubart et al. 2023).

In the Iberian Peninsula, the blue crab was first recorded in 1967 in the Tagus Estuary (Portugal), followed by sporadic records and not reaching established populations in this region (Encarnação et al. 2022). It was not until 2012 that the species was successfully established in the Ebro Delta (Castejón and Guerao 2013) and, since 2017, it has expanded into the Atlantic Ocean, reaching the Gulf of Cadiz (González-Ortegón et al. 2022). The latter study detected an inversion in the frequencies of the dominant haplotypes across the Strait of Gibraltar, linked to oceanographic barriers, such as the Almeria-Oran front, further supporting genetic structuring through the species' westward expansion (González-Ortegón et al. 2022, Schubart et al. 2023).

Of particular interest in this context is the elevated haplotypic diversity recently reported along both the Sicilian coast (28 specific haplotypes) (Mancuso et al. 2025) and the Atlantic and Mediterranean coasts of Morocco (24 haplotypes) (Lamkhalkhal et al. 2026). While part of this diversity has been linked to functional adaptation and localised genetic diversification, the dominant haplotypes consistently reflect affinities with haplotypes from lineage 1, reinforcing North America as the principal source of introduction in Europe (Schubart et al. 2023).

Two Moroccan haplotypes suggest independent introduction events into the eastern Atlantic from the North American lineage (Lamkhalkhal et al. 2026). However, correspondence between newly-detected and reference haplotypes was not reported, limiting comparability and leaving key gaps in our understanding of the introduction history of *C. sapidus* across the invaded range. These independent introduction events to the eastern Atlantic are to be expected, given its geographical position between the native and invaded range. Nevertheless, whether the Iberian/North African Atlantic populations either result from stepwise expansion from the Mediterranean or incorporate variants derived from independent introduction events remains unclear.

To improve the understanding of the invasion history of Atlantic populations, it is essential to perform multi-year genetic characterisation in areas with high propagule pressure, such as the Guadalquivir Estuary (Iberian Península, Spain) (Nehring 2011, González-Ortegón et al. 2020, Schubart et al. 2023).

The aims of this study were: 1) to characterise the genetic diversity of the invasive blue crab population in the Guadalquivir Estuary (southwest Iberian Peninsula) over a four-year period, thereby increasing the temporal sampling effort in the area and 2) to compare the detected haplotypes with reference haplotypes from both native and invaded regions.

## Material and methods

The study was conducted in the Guadalquivir Estuary (36°47.99' N 6° 20.44' W) (Gulf of Cadiz, Spain), the first recorded population in the southwest Iberian Peninsula (González-Ortegón et al. 2022, Ortega-Jiménez et al. 2024). A total of 100 mtDNA COI sequences from the Guadalquivir Estuary were obtained during six different sampling events, from the year 2021 to 2024: Two sampling events in 2021: n = 15 and n = 12, two in 2022: n = 35 and n = 11, one in 2023: n = 6, and one in 2024: n = 21 (https://doi.org/10.15470/1biece)

Genomic DNA extraction protocol, PCR conditions and primers used, as well as sequence management and comparisons of sequences obtained with GenBank and BOLD databases, follow the methodology previously explained in Cuesta et al. (2024).

The results enabled accurate matching with previously known and deposited haplotypes of this species. Newly-obtained sequences for the less frequent haplotypes have been deposited in GenBank under the accession numbers PV382972 and PV382973.

To describe the genetic structure of the blue crab population in the Guadalquivir Estuary and compare it with previous studies, the total and per sampling event haplotype richness and frequency were estimated. A Pearson's chi-squared test of independence was used to evaluate whether haplotype composition differed amongst sampling events. Given the low expected frequencies associated with rare haplotypes, p-values were estimated using the Monte Carlo simulation with 10,000 replicates. All statistical analyses and figures were produced using the R environment (R Core Team 2020).

## Results

A total of four COI haplotypes were identified in the blue crab population of the Guadalquivir Estuary during the whole study, two more than those already reported (CSWM1 and CSWM2) in previous analyses of other populations from the Gulf of Cadiz monitored in 2021 (Table 1). The two new haplotypes registered in this study, appeared with a total frequency below 1% each for the whole study (Table 1) and were a rare haplotype 1 (RH1) (PV382973) that matches 100% with 13 sequences from megalopae from the Gulf of Mexico.

These sequences were obtained and deposited in GenBank by Grey and Franco (unpublished) and correspond with MK894975-MK894977, MK894985 and MK894986 from Grand Isle (Louisiana, USA) and MK894916, MK894917, MK894920, MK894922-MK894926 from Apalachicola (Florida, USA) and a haplotype (PV382972) that matches 100% with haplotype H18 (658 bp, MG462250) described by Windsor et al. (2019). It was obtained from a specimen (CCDB1680) from the “Estuário de São Vicente”, São Paulo (Brazil), also sequenced by Zupolini et al. (unpublished) and deposited in GenBank (651 bp, JX123452). Although this haplotype H18 has been reported only from Brazil, it belongs to lineage 1, as well as two other exceptions from Brazil: H34 (ULLZ4293 from Ubatuba, São Paulo, MH985983) and H96 (CCDB994a from Laguna, Santa Catarina, MG462247) (see Windsor et al. (2019)).

The most frequent haplotypes in the Guadalquivir Estuary during the whole study were CSWM1 (66%) and CSWM2 (32%) and no significant differences in haplotype composition were detected amongst sampling events (X^2^ = 21.909; Degree of freedom (df) = NA; *p* = 0.1341) amongst the six sampling events (Fig. 1). In most of the cases, CSWM1 was the dominant haplotype, but in the second sampling event, the frequency of CWSM1 (42%) was slightly lower than the haplotype CWSM2 (58%) (Table 1, Fig. 1). The rare haplotype 1 (RH1) was detected in the first sampling event with a frequency of (7%) in 2021, while the rare haplotype 2 (H18) appeared in the fourth sampling event with a frequency of (9%) in 2022.

## Discussion

The detection of two previously unreported haplotypes in the Guadalquivir Estuary (Gulf of Cadiz, SW Spain), combined with the elevated haplotypic diversity along the coast of Morocco, raises new questions about the invasion history of the blue crab in the eastern Atlantic (Lamkhalkhal et al. 2026). Previous studies concluded that the blue crab invasion to the Gulf of Cadiz most likely shows an east-to-west expansion (González-Ortegón et al. 2022, Schubart et al. 2023), similar to that of Lessepesian species recently reported in the Guadalquivir Estuary, such as *Portunus segnis* (Forskål, 1775) (De Carvalho-Souza et al. 2023) or Alpheus cf. crassimanus Heller, 1862 (Cuesta et al. 2024, Rodríguez-Ruiz et al. 2026).

More specifically, the reduced genetic diversity, dominated by the two lineage 1 haplotypes, CSWM1 and CSWM2 (H1 and H2 in Windsor et al. (2019), respectively), suggests that the blue crab underwent a founder effect associated with the westward expansion from eastern Mediterranean populations (González-Ortegón et al. 2022, Schubart et al. 2023, Besbes et al. 2024). In accordance with this hypothesis, the inversion in the haplotypic frequency found between west Mediterranean and Atlantic invaded populations suggested a genetic barrier, such as the Almeria–Oran Front, shaping blue crab structure across the Strait of Gibraltar (González-Ortegón et al. 2022).

Our results also support the hypothesis that invaded Atlantic populations originated from Mediterranean populations. The dominance of CSWM1 and CSWM2 in the Guadalquivir Estuary, together with the inversion of their relative frequencies for most of the sampled years compared with western Mediterranean populations (González-Ortegón et al. 2022), is consistent with this invasion scenario.

In the case of the blue crab, the westward expansion has been associated with larval dispersal (Marchessaux et al. 2023), adult swimming and human-mediated transport via maritime traffic (González-Ortegón and Moreno-Andrés 2021, González-Ortegón et al. 2022).

Although our study agrees with the proposed westward expansion, the detection of two lineage 1 haplotypes, previously reported from the Gulf of Mexico and Tunisia, as well as Brazil, suggests alternative introduction pathways from the American continent contributing to the invasion history of the blue crab in the eastern Atlantic (Kolbe et al. 2008, Nehring 2011, Besbes et al. 2024). Similarly, these potential alternative introduction pathways have been recently proposed, from North America to the Atlantic Moroccan coast (Lamkhalkhal et al. 2026). Furthermore, the presence of these rare haplotypes from the beginning of the monitoring period and their persistent low frequencies in the Guadalquivir Estuary suggest that they were introduced during the early stages of the invasion, rather than representing recent introduction events (Dlugosch and Parker 2008, Sherpa and Després 2021).

However, we cannot rule out the possibility that the newly-detected rare haplotypes in this study arrived alongside the two dominant haplotypes, CSWM1 and CSWM2, from the Mediterranean Sea. This hypothesis agrees with a recent study in Tunisia (Besbes et al. 2024), in which three haplotypes were detected: CSWM1, CSWM2 and a third rare haplotype closely related to and sharing the same phylogenetic origin as one of the rare haplotypes detected in the present study. This rare haplotype from Tunisia was similar to that reported by Grey and Franco (unpublished) from three megalopae from the Gulf of Mexico: MK894943 from Pensacola (Florida, USA) and MK894962 and MK894964 from Galveston (Texas, USA). The small sample size analysed in Tunisia precludes robust conclusions regarding haplotype frequencies (Muirhead et al. 2008, Öztürk et al. 2020). However, the detection of this rare American haplotype showing 99% similarity to the one found in the Guadalquivir Estuary at the earliest sampling period of this study suggests that it could have already occurred in Mediterranean populations, but remain largely undetected (Stepien et al. 2019). This interpretation is further supported by the relatively small sample sizes typically used in genetic population studies of the blue crab across the Mediterranean (Ren et al. 2016, Schubart et al. 2023), which may have prevented these low-frequency haplotypes from being detected so far.

Furthermore, our results are consistent with the previously observed patterns of genetic flow between these two regions: from west to east for an invasive fish species, associated with the Azores current (Cunha et al. 2022) and from east to west for an invasive crab species, associated with the weaker Alboran counter-current (Ojeda et al. 2022). However, our results do not provide direct evidence of these patterns of genetic flow.

Our study supports the previously proposed westward expansion and genetic structuring between Atlantic and Mediterranean invaded populations. Additionally, the two newly-detected haplotypes raise the possibility that independent introductions from both the Gulf of Mexico and South America may also have contributed to the genetic diversity of populations in the south-western Iberian Peninsula.

## Figures and Tables

**Figure 1.**
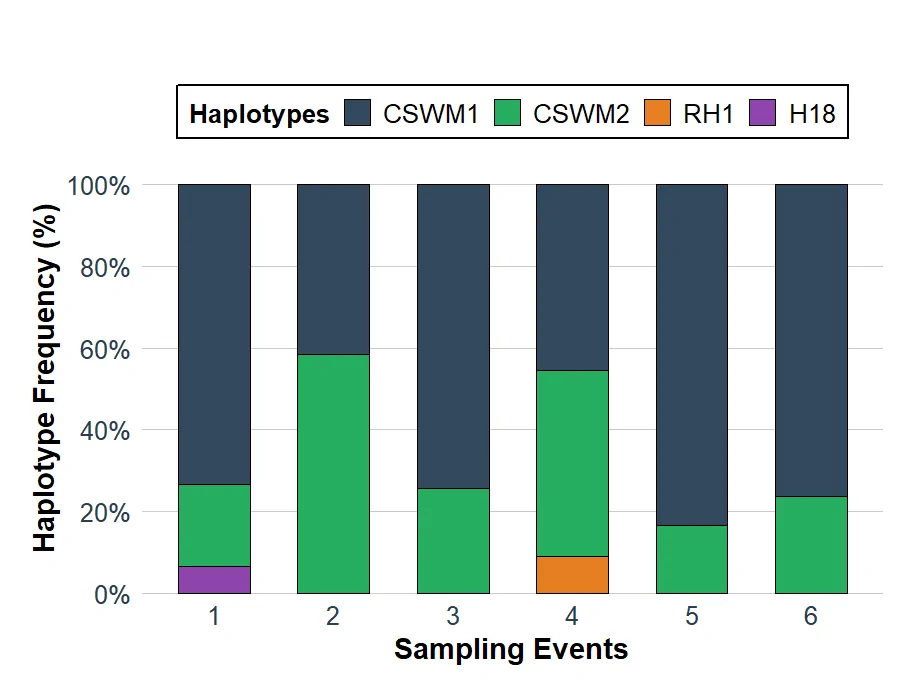
Haplotype frequency of the blue crab *Callinectes sapidus* population in the Guadalquivir Estuary at six sampling events, during the years 2021 to 2024.

**Table 1.** Total haplotype frequency (1-100%) of the four different sequenced haplotypes detected in the *Callinectes sapidus* Guadalquivir population and for each sampling event. The total number of samples (n), year and haplotypic richness (R) are also recorded at each sampling event. Haplotypes = CSWM1, CSWM2, rare haplotype 1 (RH1) and rare haplotype 2 (H18).

Sampling Event	n	Year	R	Haplotype frequency (%)			
				CSWM1	CSWM2	RH1	H18
1	15	2021	3	73	20	7	0
2	12	2021	2	42	58	0	0
3	35	2022	2	74	26	0	0
4	11	2022	3	46	46	0	9
5	6	2023	2	83	16	0	0
6	21	2024	2	76	24	0	0
Total	100		4	66	32	1	1

## References

[B14408218] Besbes N., Reale S., Scibetta S., Piazza C., Lo Monaco D., Bella C., Sadok S. (2024). Molecular barcoding identification of the invasive blue crabs along Tunisian coast. Fishes.

[B14408230] Castejón D., Guerao G. (2013). A new record of the American blue crab, *Callinectes
sapidus* Rathbun 1896 (Decapoda: Brachyura: Portunidae), from the Mediterranean coast of the Iberian Peninsula. BioInvasions Records.

[B14408239] Cuesta J. A., Acosta-Morillas V., González G., Carvalho-Souza G. F., González-Ortegón E. (2024). First record of the snapping shrimp Alpheus
cf.
lobidens (Decapoda, Caridea, Alpheidae) in the Gulf of Cadiz, an unexpected introduction to European waters from the Persian Gulf. Crustaceana.

[B14408249] Cunha R. L., Faleh A. B., Francisco S., Šanda R., Vukić J., Corona L., Dia M., Glavičić I., Kassar A., Castilho R., Robalo J. I. (2022). Three mitochondrial lineages and no Atlantic-Mediterranean barrier for the bogue *Boops
boops* across its widespread distribution. Scientific Reports.

[B14408265] De Carvalho-Souza GF,, Cuesta JA, Arana D, Lobato C, González-Ortegón E. (2023). Westward range expansion of the blue swimmer crab *Portunus
segnis* (Forskål, 1775. into Atlantic European waters. BioInvasions Records.

[B14408293] Dlugosch K. M., Parker I. M. (2008). Invading populations of an ornamental shrub show rapid life history evolution despite genetic bottlenecks. Ecology letters.

[B14408302] Encarnação J., Krug L. A., Teodósio M. A., Morais P. (2022). Coastal counter currents increase propagule pressure of an aquatic invasive species to an area where previous introductions failed. Estuaries and Coasts.

[B14408311] Enzenroß R., Enzenroß L., Bingel F. (1997). Occurrence of blue crab, *Callinectes
sapidus* (Rathbun, 1896) (Crustacea, Brachyura) on the Turkish Mediterranean and the adjacent Aegean coast and its size distribution in the bay of Iskenderun. Turkish Journal of Zoology.

[B14408329] Geller J. B., Darling J. A., Carlton J. T. (2010). Genetic perspectives on marine biological invasions. Annual Review of Marine Science.

[B14408361] González-Ortegón E., Jenkins S., Galil B. S., Drake P., Cuesta J. A. (2020). Accelerated invasion of decapod crustaceans in the southernmost point of the Atlantic coast of Europe: A non-natives’ hot spot?. Biological Invasions.

[B14408338] González-Ortegón E., Moreno-Andrés J. (2021). Anthropogenic modifications to estuaries facilitate the invasion of non-native species. Processes.

[B14408347] González-Ortegón E., Berger S., Encarnação J., Chairi H., Morais P., Teodósio M. A., Oliva-Paterna F. J., Schubart C. D., Cuesta J. A. (2022). Free pass through the pillars of Hercules? Genetic and historical insights into the recent expansion of the Atlantic blue crab *Callinectes
sapidus* to the west and the ast of the Strait of Gibraltar. Frontiers in Marine Science.

[B14408371] Holthuis L. B. (1961). Report on a collection of Crustacea
Decapoda and Stomatopoda from Turkey and the Balkans. Zoologische Verhandelingen.

[B14408380] Kolbe J. J., Larson A., Losos J. B., Queiroz K. (2008). Admixture determines genetic diversity and population differentiation in the biological invasion of a lizard species. Biology Letters.

[B14408389] Lamkhalkhal A., Vanhove M. P., Rahmouni I., Selfati M., Bazairi H., Kmentová N. (2026). Population genetic structure of the Atlantic blue crab *Callinectes
sapidus* indicates multiple introductions into Moroccan coastal lagoons. Estuarine, Coastal and Shelf Science.

[B14408400] Locci C., Azzena I, Pascale N., Ciccozzi A, Deplano I, Giantsis IA, Papadopoulos DK, Lattos A, Orru F, Puzzi CN, Scarpa F, Casu M, Sanna D. (2024). A sister species for the blue crab, *Callinectes
sapidus*? A tale revealed by mitochondrial DNA. Life.

[B14408418] Mancinelli G., Carrozzo L., Costantini M. L., Rossi L., Marini G., Pinna M. (2013). Occurrence of the Atlantic blue crab *Callinectes
sapidus* Rathbun, 1896 in two Mediterranean coastal habitats: Temporary visitor or permanent resident?. Estuarine, Coastal and Shelf Science.

[B14408429] Mancuso M., Tiralongo F., Calogero G. S., Manganaro G., Nota A., Ferrito V., Pappalardo A. M. (2025). Evidence of positive selection in the COI mitochondrial gene in the blue crab *Callinectes
sapidus* (Decapoda: Portunidae). Scientific Reports.

[B14408441] Marchessaux G., Chevalier C., Mangano M. C., Sarà G. (2023). Larval connectivity of the invasive blue crabs *Callinectes
sapidus* and *Portunus
segnis* in the Mediterranean Sea: A step toward improved cross border management. Marine Pollution Bulletin.

[B14408450] Muirhead J. R., Gray DK, Kelly DW, Ellis SM, Heath DD, Macisaac HJ. (2008). Identifying the source of species invasions: sampling intensity vs. genetic diversity. Molecular Ecology.

[B14408461] Nehring S., Galil B. S. (2011). In the wrong place-alien marine crustaceans: distribution, biology and impacts.

[B14408474] Ojeda V., Serra B., Lagares C., Rojo-Francàs E., Sellés M., Marco-Herrero E., Garia E., Farré M., Arenas C., Abelló P., Mestres F. (2022). Interannual fluctuations in connectivity among crab populations (*Liocarcinus
depurator*) along the Atlantic-Mediterranean transition. Scientific Reports.

[B14408490] Ortega-Jiménez E., Cuesta JA, Laiz I., González-Ortegón E. (2024). Diet of the invasive Atlantic blue crab *Callinectes
sapidus* Rathbun, 1896 (Decapoda, Portunidae) in the Guadalquivir estuary (Spain). Estuaries and Coasts.

[B14408499] Öztürk R. Ç., Terzi Y., Feyzioğlu A. M., Şahin A., Aydın M. (2020). Genetic characterization of the invasive Blue crab, *Callinectes
sapidus* (Rathbun, 1896), in the Black Sea. Regional Studies on Marine Science.

[B14408509] Team R Core (2020). R: A language and environment for statistical computing.

[B14408517] Ren G., Ma H., Ma C., Wang W., Chen W., Ma L. (2016). Genetic diversity and population structure of *Portunus
sanguinolentus* (Herbst, 1783) revealed by mtDNA COI sequences. Mitochondrial DNA Part A.

[B14408528] Rodrigues M. A., Dumont L. F.C., Santos C. R.M., D'Incao F., Weiss S., Froufe E. (2017). Two distinct mtDNA lineages of the blue crab reveal large-scale population structure in its native Atlantic distribution. Estuarine, Coastal and Shelf Science.

[B14408539] Rodríguez-Ruiz Á., León-Cobo M. J., Cuesta J. A., Carvalho-Souza G. F., González-Gordillo J. I., González-Ortegón E. (2026). Life-history traits linked to invasion: Reproduction and larval dynamics of an invasive snapping shrimp at the southernmost coast of Europe. Estuarine, Coastal and Shelf Science.

[B14408559] Schubart C. D., Deli T., Mancinelli G., Cilenti L., Gil Fernández A., Falco S., Berger S. (2023). Phylogeography of the Atlantic blue crab *Callinectes
sapidus* (Brachyura: Portunidae) in the Americas versus the Mediterranean Sea: Determining origins and genetic connectivity of a large-scale invasion. Biology.

[B14408571] Sherpa S., Després L. (2021). The evolutionary dynamics of biological invasions: A multi‐approach perspective. Evolutionary Applications.

[B14408550] Stepien CA,, Snyder MR., Elz AE. (2019). Invasion genetics of the silver carp *Hypophthalmichthys
molitrix* across North America: Differentiation of fronts, introgression, and eDNA metabarcode detection. PLOS One.

[B14408589] Windsor A. M., Moore MK, Warner KA, Stadig SR, Deeds JR. (2019). Evaluation of variation within the barcode region of cytochrome C oxidase I (COI) for the detection of commercial *Callinectes
sapidus* Rathbun 1896 (Blue crab) products of non-US origin. PeerJ.

